# Choroid Plexus in Alzheimer’s Disease—The Current State of Knowledge

**DOI:** 10.3390/biomedicines10020224

**Published:** 2022-01-21

**Authors:** Tiago Gião, Tiago Teixeira, Maria Rosário Almeida, Isabel Cardoso

**Affiliations:** 1 Molecular Neurobiology Group, i3S-Instituto de Investigação e Inovação em Saúde, Universidade do Porto, Rua Alfredo Allen 208, 4200-135 Porto, Portugal; tteixeira@i3s.up.pt (T.T.); ralmeida@ibmc.up.pt (M.R.A.); 2 IBMC—Instituto de Biologia Molecular e Celular, Universidade do Porto, Rua Alfredo Allen 208, 4200-135 Porto, Portugal; 3 Departamento de Biologia Molecular, ICBAS—Instituto de Ciências Biomédicas Abel Salazar, 4050-013 Porto, Portugal; 4 Faculdade de Medicina, Universidade do Porto, 4200-319 Porto, Portugal

**Keywords:** choroid plexus, blood–cerebrospinal fluid barrier, Alzheimer’s disease, neurodegenerative disorder, barrier disruption, novel therapies

## Abstract

The choroid plexus (CP), located in each of the four ventricles of the brain, is formed by a monolayer of epithelial cells that surrounds a highly vascularized connective tissue with permeable capillaries. These cells are joined by tight junctions forming the blood–cerebrospinal fluid barrier (BCSFB), which strictly regulates the exchange of substances between the blood and cerebrospinal fluid (CSF). The primary purpose of the CP is to secrete CSF, but it also plays a role in the immune surveillance of the central nervous system (CNS) and in the removal of neurotoxic compounds from the CSF. According to recent findings, the CP is also involved in the modulation of the circadian cycle and neurogenesis. In diseases such as Alzheimer’s disease (AD), the function of the CP is impaired, resulting in an altered secretory, barrier, transport, and immune function. This review describes the current state of knowledge concerning the roles of the CP and BCSFB in the pathophysiology of AD and summarizes recently proposed therapies that aim to restore CP and BCSFB functions.

## 1. Introduction

The central nervous system (CNS) has barriers with selective permeability that are essential to maintain brain homeostasis. The blood–brain barrier (BBB), which spans nearly the whole vasculature of the brain and controls blood–brain transport, is the most notable example. Within the ventricles, the choroid plexus (CP) provides an interface between the cerebrospinal fluid (CSF) and the blood, establishing the blood–cerebrospinal barrier (BCSFB). The CP is a highly vascularized structure comprising a monolayer of epithelial cells with microvilli that surrounds connective tissue and permeable capillaries [1]. The CP assists in the influx of molecules from the blood, as well as in the removal of toxic substances and metabolites from the CSF. Despite its recognized involvement in the homeostasis of the CNS, the BCSFB has always been belittled by the scientific community when compared to the BBB, and, together with the CP, has remained outside the sphere of interest of neuroscientists. In recent years, several works have reported CP structural and functional changes that have implications in the pathophysiology of many brain diseases, bringing interest to the subject [2]. Changes in CP secretome, as well as the presence of harmful molecules, also change the composition of CSF. As a result, and because changes in the CSF are frequently a mirror of a specific pathological scenario, detecting CSF biomarkers has diagnostic value. Furthermore, the CP is in close contact with the subventricular zone, along the lateral wall of the lateral ventricle, which is crucial in neurogenesis, indicating that the CP–CSF interaction may play a major role in neurodevelopmental diseases [3].

## 2. The Structure and Cell Organization of the Choroid Plexus

The brain has four interconnected cavities, known as ventricles, that are filled with CSF. The CP, a veil-like structure that floats in the CSF, is located within the ventricles. Structurally, the CP is composed of a single layer of cuboidal epithelial cells settled on a basement membrane. The epithelium has microvilli on its apical surface and basolateral folding, which considerably increases the surface area of the CP available for exchanges between CSF and blood [1]. An extensive network of anastomosed capillaries, arising from branches of the anterior and posterior choroidal arteries, penetrates the connective tissue adjacent to the basement membrane. The CP is extensively irrigated, with a blood flow 10 times higher than the cortex [4]. The CP vessels are highly permeable due to the existence of fenestrations and to the lack of tight junctions, allowing paracellular transport [2].

The transport of molecules across the epithelium occurs mostly by a strictly regulated transcellular transport since the paracellular pathway is blocked by tight junctions. Several nutrients, hormones, and peptides reach the CSF via the BCSFB, and the combined action with metabolic enzymes restricts the access of other compounds [1,5]. The high energy demands of transcellular transport across the BCSFB is made evident by the elevated mitochondria content on cell cytoplasm. These organelles account for 12–15% of the cytoplasm volume and are more enriched at the apical surface [6].

Other types of cells can be found associated to the CP, such as a niche of neuro stem cells (NSCs), which is localized in the subventricular zone of the lateral ventricle with processes that contact with the ependymal layer in order to interact with CSF factors and develop into either neurons or oligodendrocytes [7].

There are also resident macrophages in the BCSFB, the Kolmer cells (epiplexus cells) and the stromal macrophages, which reside in the apical surface of the CP epithelium and in the stroma, respectively. The Kolmer cells, which derive from circulating monocytes, have an ameboid shape and have a transcriptional expression resembling activated microglia, while the stromal macrophages have a more stellate shape and an expression profile similar to perivascular and meningeal macrophages [8,9,10].

## 3. General Physiology of the CP

The CP is the major contributor for CSF secretion, and there are several theories explaining this process. On one hand, it is hypothesized that through energy-dependent transport, by means of ATP-binding cassette (ABC) transporters, an ion concentration gradient is established that allows the flow of water across the choroid plexus epithelial cells (CPECs) by aquaporins (or by paracellular transport); on the other hand, it was also suggested that CSF secretion is due to a hydrostatic pressure gradient between the blood and the ventricles [5]. Both cases lead to the secretion of CSF, in addition to aiding the motile cilia of ependymal cells to generate a flow that is the source of the migration of bioactive molecules, such as proteins and neurotrophic factors that are expressed in the CP, namely, transthyretin (TTR), insulin-like growth factors (IGF), and transforming growth factor-β (TGF-β) [2,11], or transported across the CP, such as thyroxine (T4) [12].

The expression of genes responsible for maintaining and integrating the mammalian circadian rhythm was also uncovered in the CP, pointing to its possible role as an extra-suprachiasmatic nucleus circadian clock, as will be mentioned further on [2]. Taste receptors and a functional taste signaling pathway have also been observed on the CP, which points to a possible chemical surveillance role of the CSF [13].

In addition to the presence of resident macrophages of the BCSFB, which were mentioned previously in this review, the CP aids in immune surveillance by producing molecules essential for leukocyte migration as well as providing T cells with an optimal proliferative environment, the stroma [14].

Finally, besides its role as a physical barrier, the CP also acts as an enzymatic barrier [15], which is especially important during brain development, while the liver is still not properly functioning [1]. This is achieved by the high expression of glutathione S-transferases, which are a family of metabolic isoenzymes with the ability to catalyze the conjugation of glutathione with xenobiotics substrates and reactive molecules with the purpose of detoxification and to protect cells from oxidative damage [16].

## 4. BCSFB and BBB Comparison

Despite their common denomination as barriers, the BBB and the BCSFB have structural and cellular differences and even distinct ontogeny [17,18].

The BCSFB is composed of the endothelial cells of the capillaries, the stroma, the CP epithelium, and the ependymal cells. The BCSFB capillaries have fenestrations, meaning that, unlike the BBB capillaries, which are tightly connected, blood exchanges can occur more easily. However, these molecules do not freely reach the brain due to the barrier properties of the CPECs. These cuboidal epithelial cells arranged in a monolayer are connected by tight junctions (TJ), adherent junctions (AJ), and gap junction (GJ) [19]; nevertheless, these are different from the ones found in the BBB as the TJs of the BCSFB are shorter and in higher number on the apical portion of the tissue [11].

Conversely, the BBB is composed by the neurovascular unit (NVU) which is comprised by the endothelial cells of the capillaries, mural cells (the pericytes and vascular smooth muscle), the perivascular space, astrocytes, and microglia [20].

**Figure 1:**
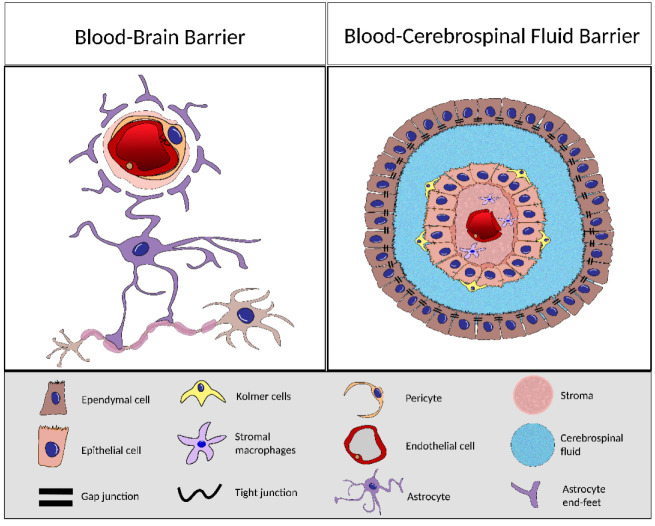
Schematic representation of the blood–brain barrier (BBB) and blood–cerebrospinal fluid barrier (BCSFB), depicting the cellular constituents of each barrier.

The endothelial cells of the BBB capillaries are connected by the TJ, which limit the exchanges with the brain. The perivascular space is a region in the BBB where the exchanges between the brain and the blood occur and is crucial for clearance of brain waste and neuron metabolism products [20] (Figure 1).

Whilst both the BBB and the BCSFB act as physical barriers, they display different resistance to the passage of molecules, which is evidenced by the different values of transendothelial electrical resistance (TEER). Experiments with cell lines have defined the TEER of the BBB as ~1800 Ω cm^2^ [21,22] and that of the BCSFB as ~270 Ω cm^2^ [23], which is still higher than the remaining tissues where the TEER is around 3–33 Ω cm^2^ [24]. These values are consistent with the previously mentioned characteristics of the TJs of each barrier since the BBB has higher proximity and tighter paracellular spaces, thus implying a higher resistance to current.

The cover area of each barrier is also a major difference, although there is not a consensus on its relation. It has been reported that the total surface area of the BBB is about 20 m^2^ while that of the BCSFB is about 5000 times smaller, between 0.004 and 0.02 m^2^ [25]. However, in a 1990 experiment, R.F. Keep et al. [26] used electron microscopy and determined that the surface area per cell per weight of brain in the rat was about 75 cm^2^. Assuming 155 cm^2^ of area for the rat BBB, these results suggest that the BCSFB has approximately half the surface area of the BBB, substantially larger than the value previously mentioned. Moreover, R.F. Keep did not consider the microvilli, whose main function is the increase of the available exchange surface, in the total area.

## 5. Aging of the BCSFB and Disease-Induced Alterations

Despite its important roles, the CP is very susceptible to modifications in a diversity of disease scenarios and even in normal physiological aging, which causes significant alterations in its functions that will be briefly mentioned in the following section.

In normal physiological aging, the CP undergoes morphogenic changes (the basement membrane becomes thicker, the epithelial cells flatten and become shorter, and the nuclei become irregular) and several inclusions begin to appear, such psammoma bodies (calcium inclusion in the stroma), lipofuscin deposits, and Biondi rings. These inclusions will be discussed with more detail further on. The secretory and regulatory functions of the CP are also altered as the expression of energy-associated proteins and enzymes is diminished. In the aging rat, the expression of proteins involved in CSF secretion has also been found downregulated, which could explain the reduced CSF secretion observed in elderly humans [27,28]. Additionally, the expression of TTR has also been found to be altered with age, although there is not a consensus whether it is increased or decreased [29,30].

BCSFB alterations are also present in diseases such as amyotrophic lateral sclerosis (ALS) in which patients display elevated total protein levels and increased blood proteins in the CSF, indicating that there may be disruption of the barrier and/or decreased clearing, which was also confirmed by transcriptomic and immunohistochemistry studies where the expression and patterns of claudin-5 and zonula occludens-1, respectively, were downregulated [31].

In multiple sclerosis (MS) patients, there are reports of claudin-3 dysregulation, which leads to increased leukocyte infiltration due to the disruption of the BCSFB further enhancing the causes and symptoms of the disease [32].

BCSFB disruption can also be observed after an ischemia-reperfusion incident; however, there are reports that imply that there is also a partial recovery 24 h after the insult. This disruption may occur due to the peripheral signaling, and the CP also promotes tissue repair by the expression of growth factors [33].

Through mechanical modelling, researchers have hypothesized that there are alterations in the BCSFB that lead to increased permeability to sodium, explaining its higher concentration in the CSF of migraine patients [34]. The BCSFB and the CP can even suffer alterations in major depression disorder (MDD), where the expression of TTR, which is the main secreted protein by the CP, is reduced due to lower activation of the 5-HT_2C_ serotonin receptor [32,35].

The access of pathogens to the brain via the BBB is extensively established, but there is also clear evidence of pathogen entrance into the CNS via the BCSFB. Bacteria, viruses, fungi, and parasites can use a variety of strategies to penetrate cellular barriers. They can either use transcellular transport to cross the cell, or disrupt TJs for a paracellular mechanism and potentially hijack infected phagocytic host cells and use a “Trojan horse” method to enter the brain [8]. Moreover, pathogens can cause inflammatory responses in the brain after breaching these barriers, eventually leading to cerebral inflammation. When BCSFB integrity is compromised, cytokines and leukocytes enter the CSF, leading to the activation of microglia, the resident immune cells of the brain.

## 6. Alzheimer’s Disease and the CP

The average life expectancy has increased dramatically over the past few decades. As a result, the growing number of elderly people in the world’s population has fueled interest in researching age-related diseases.

Alzheimer’s disease (AD), which has age as the primary risk factor, stands out in this group. At the clinical level, this neurodegenerative disorder is characterized by progressive memory and cognitive dysfunction. Specific brain lesions known as AD hallmarks are found in patients’ brains. One of them is the occurrence of neurofibrillary tangles in the cytoplasm of neurons caused by Tau protein hyperphosphorylation. Another is the presence of senile plaques on the extracellular space due to the aggregation and deposition of the amyloid-β peptide (Aβ) [36]. The imbalance between Aβ generation and its clearance correlate with the accumulation of this peptide. It has been proposed that Aβ peptide and its species, particularly oligomers, are the major drivers of neuroinflammation, which is also a key feature of the brain of AD subjects. In fact, both acute and chronic inflammation are associated with the cognitive decline characteristic of the pathology [37].

**Figure 2:**
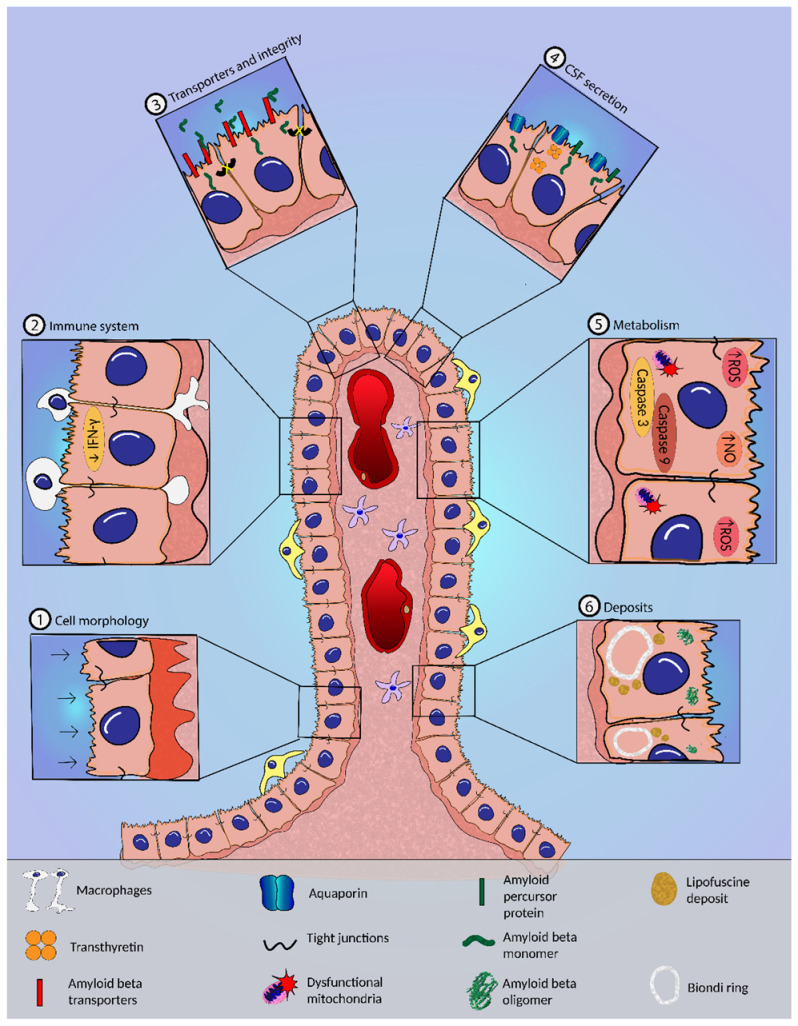
The choroid plexus (CP) and the blood–cerebrospinal barrier (BCSFB). The zoom highlights the alterations occurring in AD: (1) cell morphology—CP epithelial cell (CPEC) flattening and increased thickness and irregularity of the basement membrane; (2) immune system—impairment of leukocyte trafficking and decreased IFN-γ signaling; (3) transporters and integrity—tight junction disruption and deregulation of transporters of amyloid-β peptide (Aβ); (4) CSF secretion—diminished AQP1 expression, decreased secretion of CSF proteins, and increased production of Aβ in the CP; (5) metabolism—mitochondrial dysfunction and increased nitric oxide (NO) and reactive oxygen species (ROS) levels, and caspase-3 and -9 expression; (6) deposits—deposits of Aβ, lipofuscin granules, and Biondi ring tangles accumulate in CPECs.

While the BBB, which encompasses almost all the brain’s vasculature, has been extensively studied in AD, the CP and the BCSFB have been neglected. With this in mind, the present review focuses on the CP, and how it, like other regions of the brain, undergoes structural and functional changes during aging and AD development and progression (Figure 2).

### 6.1. Morphological Alterations in the CP in AD

Several morphological changes have been described in the CP of AD patients, including flattening of epithelial cells and thickening of the irregular basement membrane, as compared to age-matched controls [38,39]. Aβ may also induce morphological changes in the CP cells, such as nucleus and cell volume shrinkage, as shown by CPECs from Aβ-injected mice [40]. Dense fibrosis of the underlying connective tissue is also present, which could be related to the increased collagen IV content, reported later [38,39]. Biondi ring tangles, which are intracellular inclusions, were observed to be more prevalent in AD patients when compared to control individuals [41,42]. Despite the discovery of Biondi body-like inclusions in an elderly chimpanzee, these inclusions were only ever detected in aged human CP, making their study difficult [43]. Histological analysis revealed several proteins constituting these aggregates, including tau protein, fibronectin, ubiquitin, and P component, as well as the presence of lipid droplets. The occurrence of these structures in the cytoplasm can cause mechanical damage to the plasma membrane [41,42,44]. Lipofuscin granules, which arise from highly oxidized cross-linked macromolecules and affect vesicle trafficking and cellular physiology, are also found in the cytoplasm of CPECs from aged and AD mice [38,42,45].

The CP of AD patients and mouse AD models are also characterized by deposits of Aβ [39,46,47] that may disrupt several CP functions. However, the alterations described are not exclusive of the AD brain and have been reported in aged mice [39,48]. Nevertheless, Aβ seems to play a crucial role in the degeneration of the biochemical pathways of the brain, including in the CP, as detailed in the sections below.

### 6.2. CSF Dynamics and Secretion in AD

Maintenance of the composition and volume of the CSF is essential to ensure normal brain function. The CP assists in the removal of harmful compounds from the CSF [49].

For instance, Aβ must be continuously removed from the brain to prevent its accumulation and aggregation. In this process, Aβ from the brain parenchyma easily reaches the CSF and flows to the CP vicinity to be transported out [50]. However, there is a dramatic alteration in CSF dynamics of AD patients, as evidenced by the decline in the CSF turnover and production [51,52]. Augmented ventricular volume also occurs in AD patients, as a consequence of the decreased neuronal mass, which contributes to the low turnover [52]. There also appears to be increased resistance to CSF absorption, as indicated by the elevated CSF pressure [53]. In aged mice, Aβ begins to accumulate before there is any reduction in CSF production and turnover, suggesting that the accumulation of the peptide is not a consequence of the decreased CSF turnover [54].

The transport of water to the CSF is carried out primarily through the aquaporin-1 water channel (AQP1), which is mainly located on the apical side of the epithelial cell membrane [55]. Consistent with the low CSF turnover reported above, in AD, this channel has its levels lowered [39], although its gene expression is not altered [56]. Several solute transporters and associated enzymes also have altered expression in AD patients, indicating the disruption of ionic transport [56,57].

As a result of the decreased CSF turnover, there is an accumulation of harmful compounds and nutrients, preventing them from reaching the brain parenchyma [58].

The composition of CSF varies as the disease progresses, and proteins such as TTR and gelsolin, which are known as neuroprotective and produced by the CP, become reduced, due in part to the decline of this organ’s secretory abilities [39,59,60]. Microarray analysis also revealed that the vascular endothelial growth factor (VEGF) signaling pathway, an important mediator of angiogenesis and inflammation, is upregulated in the CP, in AD [61]. On the other hand, the CP has been described as being able to produce Aβ [62,63,64], and this process occurs at a faster rate in AD patients [62,64,65].

### 6.3. BCSFB Integrity in AD

Aβ increased levels in AD lead to its deposition in the CP, impacting its function and, as a result, the integrity of BCSFB. This allows unwanted molecules to be transported paracellularly, compromising CSF homeostasis. Intraventricular administration of oligomeric Aβ increased matrix metalloproteases (MMP) expression (notably the MMP-3) and downregulated tight junction proteins claudin-5, occludin, zonula occludens-1, and claudin-1 in the CP [40]. Downregulation of claudin-5, claudin-11, and claudin-18 is also found in AD patients [56,57]. The CP of AD patients and of an AD mouse model also presented increased MMP-9 levels, which co-localized with Aβ deposits, and lower levels of the tight junction protein zonula occludens-1 [47]. MMP3-deficient mice treated with oligomeric Aβ elucidated the role of these MMPs in BCSFB integrity, as these animals had less BCSFB leakage than control mice [40]. These findings support the notion that in AD, largely because of Aβ peptide, there is a decline in tight junction proteins and an increase in MMP levels in the CP that may compromise BCSFB integrity and function.

### 6.4. Transport of Aβ and Other Compounds across the BCSFB in AD

CPECs are able to carry Aβ from the CSF side to the blood side and vice versa, with efflux of the peptide into the bloodstream being favored [66]. Several classic Aβ transporters at the BBB are also involved in the clearance of Aβ through the BCSFB in the CP epithelium. The low-density lipoprotein receptor-related protein 1 (LRP-1), low-density lipoprotein receptor-related protein 2 (LRP-2), and P-glycoprotein (P-gp) are the primary carriers of Aβ to the bloodstream via BBB and BCSFB. In contrast, the receptor for advanced glycation end products (RAGE) facilitates the entry of Aβ into the CSF. However, in AD mice, the expression of LRP-1 and RAGE was found to be increased in the BCSFB [39,61]. Transcriptomic analysis confirms the upregulation of LRP-1 at the CP of AD patients [61]. On the other hand, no Aβ transporters were detected in CP vessels, which is reasonable since the high permeability of the vessels would render them useless [39]. A transcriptome analysis found that older rats had higher expression of LRP-1 and P-gp at the BCSFB, but no variations in RAGE expression, when compared to younger rats [67]. BBB LRP-1 and P-gp decline with age, and notably in AD, and there is a strong negative correlation between the expression of LRP-1 on vessels and regional Aβ accumulation, implying that the presence of Aβ impacts this transporter [48,68,69]. It is possible that the BCSFB attempts to compensate for the loss of Aβ transporters at the BBB, increasing their levels and restoring some efflux capability, although this remains to be determined.

The accumulation of Aβ, on the other hand, appears to impair the LRP-2-mediated transport across the epithelium of proteins such as leptin, albumin, and TTR [46]. Since these last two are recognized Aβ carriers, it is possible that a decrease in their levels in the CSF further exacerbates the pathology. The LRP-2 decline also affects IGF-I influx, a significant neuroprotective protein in AD [70,71].

### 6.5. Metabolic Alterations and Oxidative Stress in the CP in AD

Oxidative stress, as one of the earliest events in AD pathogenesis, plays a significant role in disease development [72]. Studies with AD subjects and with AD transgenic mice demonstrated that Aβ induces nitric oxide (NO) generation and increases reactive oxygen species (ROS) and CPEC death, as evidenced by increased caspase-3 and -9 expression [47]. The presence of oxidation markers in different proteins in the CP of late-stage AD patients as a result of increased reactive oxygen species may affect CP function [73].

According to a large-scale gene expression analysis, AD patients are characterized by an upregulation of the unfolded protein response, endoplasmic reticulum stress pathway, and the protein ubiquitin pathway, which is the reflex of the increased cellular stress [56,74]. The glutathione-mediated detoxification pathway and the urea cycle, on the other hand, were found to be downregulated in the CP, suggesting that a sink action could be impaired in AD [56].

The mitochondrial energy metabolism is also impaired in CP of AD patients, as seen by the alteration of the activity and assembly of mitochondrial respiratory chain complexes I and IV [47,75]. In addition, mitochondrial ATP synthase, which is needed for ATP synthesis, is downregulated in these patients [57].

### 6.6. Inflammation and CP in AD

The CP also performs immune surveillance of the CNS, acting as a gateway for leukocyte entry into the brain parenchyma. It constitutively expresses adhesion molecules and chemokines, allowing leukocyte trafficking by BCSFB during immune responses [14].

One of the most prominent hallmarks of AD is the neuroinflammation that occurs as a result of a disturbance of the balance of anti-inflammatory and pro-inflammatory signaling. It is proposed that AD is characterized by an early acute inflammation phase with microglial activation in response to neurotoxic molecules that can become chronic, due to the system’s inability to mount an adequate immune response [14].

The involvement of inflammation in AD in the CP is also well documented. The expression of many genes associated with acute phase response, cell adhesion, and cytokines is elevated in the CP of an AD patient [61]. This intense immune response is attributed, in part, to a failure in the recruitment of immune cells to the brain, through the CP. The gateway activity for leukocyte trafficking was found to be disrupted in AD mouse models as a consequence of decreased CP interferon-γ (IFN-γ) signaling, which affected the induction of leukocyte trafficking determinants [76,77]. Interestingly, transcriptomic analysis has revealed that the gene encoding IFN-γ was more expressed in the CP of 3-month-old AD mice compared with non-transgenic controls. IFN-γ levels in AD mice were further decreased compared to the control group at the age of 5–6 months, which lasted until 11–12 months of age. Notably, the genes involved in type I interferon response showed an overall overexpression in AD mice at the ages studied [78].

IFN-γ signaling can be regulated by Foxp3^+^ regulatory T cells, reducing IFN-γ availability at the CP. In AD mice, the transient depletion of these cells or pharmacological inhibition of their activity leads to decreased Aβ load, reduced neuroinflammation, and improved cognitive function. This is due to increased IFN-γ in the CP, which enhances the gateway activity, resulting in the recruitment of regulatory T cells and monocyte-derived macrophages at sites of Aβ plaque formation [76]. The immune checkpoint programmed cell death protein 1 (PD-1) regulates T cell activity by suppressing it. In AD mouse models, treatment with a blocking antibody directed at PD-1 increases IFN-γ expression in the CP and starts an IFN-γ-dependent immune response [79].

The tumor necrosis factor α (TNF-α) has also been shown to promote leukocyte entry via the CP through NFκB/p65 signaling [80]. This movement of immune cells is impaired in the presence of NO, which is increased in the CP of AD patients [47]. The administration of NO scavengers to AD mice induced NFκB/p65 pathway activation and expression of CP leukocyte trafficking determinants, restoring CP gateway activity [80].

TNF was recently identified as the most significant upstream regulatory cytokine in the CP of late-stage Alzheimer’s disease patients. Tumor necrosis factor receptor-1 (TNF-R1) ablation alleviated epithelial morphological changes, reduced CP inflammation, and restored BCSFB integrity in two different AD mouse models. In animals missing TNF-R1, the observed integrity can be explained by the decreased levels of MMPs and the preservation of tight junctions [81]. Interestingly, intracerebroventricular injection of Aβ oligomers in young mice increased TNF-α gene expression in CP, as well as interleukin-6 (Il-6) and nitric oxide synthase, strengthening its involvement in the disease [40]. The genes that code for interleukin-1 receptors are also increased in AD, being associated with acute and chronic inflammation [57].

### 6.7. Features of CP Stem Cell in AD

The formation of new neurons and glia is fundamental during embryonic development and occurs also after birth and throughout adulthood in certain brain regions. During maturity, this phenomenon is found in the olfactory bulb, the granular cell layer of the hippocampus, and the ependymal membrane of the lateral ventricles (subventricular zone) [82]. It has also been suggested that some CPECs have neural stem cell characteristics, such as the ability to proliferate and express neuronal and glial markers [83]. Others went further and suggested the existence of neural progenitor cells among the epithelial cell [84].

Regarding this function in AD, in vivo and in vitro approaches showed that Aβ regulates the proliferation and differentiation of neural progenitor cells into neurons, which, however, have reduced survival [85]. This can be seen as a compensatory process, allowing the replacement of damaged and dead neurons [86].

### 6.8. Circadian Cycle Disturbed at CP in AD

Recently, the CP was implicated in the modulation of circadian rhythm, which corresponds to the daily oscillations of diverse biological processes that keep the body rhythms in synchrony with the environment’s external light–dark cycles. The hypothalamic suprachiasmatic nucleus is the master circadian clock and coordinates circadian gene expression in the peripheral clocks [87]. On the other hand, CP has also been shown to produce melatonin, probably controlled by the CP clock, and to influence the rhythm of the master clock, the suprachiasmatic nucleus [88]. The CP was recently highlighted as one of the peripheral circadian clocks, as concluded by the expression of circadian clock genes in rat CP. Circadian oscillations in the expression of these genes were less noticeable in males than in females [89], with estrogens contributing to this difference [90]. 

The circadian rhythm of CP in mouse AD is unregulated, as shown by the aberrant oscillations in the expression of clock genes. The circadian rhythmicity was recovered when a CP cell line was treated with melatonin in the presence of Aβ [91]. The same group of researchers showed the impact of circadian rhythm on the secretion of Aβ scavengers—apoliprotein J (ApoJ) and TTR—in non-transgenic rat explants, with a clear pattern of fluctuations in the expression of those proteins [92].

### 6.9. CP Epithelial Cell Implants as a Therapy in AD

The CP is associated with numerous pathophysiological processes and is crucial for the maintenance of brain homeostasis. In light of the popularity of the CP in recent years, the application of epithelial cell implants in neurodegenerative diseases as cell therapy to restore brain tissue and function has become a reality. The CP ability to secrete neuroprotective growth factors, neurotrophins, hormones, and proteins, as well as the presence of epithelial cells with neural stem cell properties, are some of the points that support this therapeutic path in AD. Strengthening this idea, co-cultures of neurons and CPECs resulted in increased neuronal survival and proliferation compared to cultures with neurons alone. These results were related to reduced Aβ and increased neprilysin levels in the conditioned media, reflecting the neuroprotective potential conferred by the CPECs [93]. Taking into account the protective role of CPECs, researchers implanted these cells in the hippocampus of AD mice. Post-mortem analysis revealed a decrease in Aβ deposits, hyperphosphorylation of tau, and astrocytic inflammation. Behavior analysis also revealed improved spatial and non-spatial memory [93]. In another study, the implantation of microcapsules with CPECs in the cortex, following intrahippocampal Aβ injection, resulted in improved memory in a rat model. Brain sample analysis also revealed that transplantation of encapsulated CPECs resulted in a significant increase in neurogenesis and antioxidant activity, combined with a decrease in apoptosis, gliosis, and neuroinflammation [94].

Although not from CP, very recent data demonstrated that transplantation of human amniotic epithelial cells and treatment with lycopene or their combination can improve learning and memory abilities and decrease Aβ deposition in an acute AD rat model. Decrease levels of proinflammatory and anti-inflammatory cytokines were also detected in CSF and hippocampus via regulation of CP, as seen by the decrease in Toll-like receptor 4 and nuclear factor-κB p65 signaling at the CP. This work highlights the immunomodulatory ability of the CP [95,96].

## 7. Conclusions

In conclusion, increasing evidence reinforces the role of the CP and its interface, the BCSFB, in the pathophysiology of AD. Changes in CSF secretion and dynamics, inflammation, oxidative stress, and BCSFB integrity and transport are reported here. These changes impair the clearance of the Aβ peptide, causing it to accumulate and exacerbating the pathology.

However, it remains to be confirmed as to whether these alterations in the CP are a cause or a consequence of the generalized neuroinflammation occurring in the AD brain, and thus further studies should be conducted.

Therapeutic approaches, such as the transplantation of CPECs in rodents, reveal promising benefits at the level of the CP and the brain in general in AD, suggesting a viable alternative treatment for AD.

## Data Availability

Not applicable.
